# Supplementary material for: Pro-Inflammatory Priming of Umbilical Cord Mesenchymal Stromal Cells Alters the Protein Cargo of Their Extracellular Vesicles
Source: Cells. 2020 Mar 16;9(3):726. doi: 10.3390/cells9030726 (PMC7140705; doi:10.3390/cells9030726)
Supplement: Supplementary file 1 [file cells-09-00726-s001.pdf]

Supplementary Materials

Supplementary Table 1: List of antibodies used to characterise UCMSCs. All antibodies were directly conjugated to a flurochrome and used in a working concentration of 5μg/ml. All antibodies were purchased from BD Biosciences.

Antibody	Conjugated Flurochrome	Dilution	Isotype Control
CD105	APC	1:20	APC Mouse IgG1
CD90	PE-CF594	1:20	PE-CF594 Mouse IgG1
CD73	BV421	1:20	BV421 Mouse IgG1
CD14	PerCP-Cy5.5	1:20	PerCP-Cy5.5 Mouse IgG2b
CD45	PE-CF594	1:20	PE-CF594 Mouse IgG1
CD34	APC	1:5	APC Mouse IgG1
CD19	BV421	1:20	BV421 Mouse IgG1
CD106	APC	1:2.5	APC Mouse IgG1
CD146	PE-CF594	1:11	PE-CF594 Mouse IgG1
HLA-DR	APC	1:5	APC Mouse IgG2b
APC Mouse IgG1	APC	1:5	
PE-CF594 Mouse IgG1	PE-CF594	1:50	
BV421 Mouse IgG1	BV421	1:100	
PerCP-Cy5.5 Mouse IgG2b	PerCP-Cy5.5	1:5	
APC Mouse IgG2b	APC	1:5	

Supplementary Table 2: List of surface marker antibodies from the MacsPlex Exosome Detection kit (Miltenyi Biotec, Woking, UK). REA and mIgG1 isotype controls were used.

	Antibody	Isotype		Antibody	Isotype
**1.**	CD3	mIgG2a	**20.**	CD25	mIgG1
**2.**	CD4	mIgG2a	**21.**	CD49e	mIgG2b
**3.**	CD19	mIgG1	**22.**	ROR1	mIgG1κ
**4.**	CD8	mIgG2a	**23.**	CD209	mIgG1
**5.**	HLA-DRDPDQ	REA	**24**	CD9	mIgG1
**6.**	CD56	REA	**25.**	SSEA-4	REA
**7.**	CD105	mIgG1	**26.**	HLA-ABC	REA
**8.**	CD2	mIgG2b	**27.**	CD40	mIgG1κ
**9.**	CD1c	mIgG2a	**28.**	CD11c	mIgG2b
**10.**	CD63	mIgG1κ	**29.**	CD81	REA
**11.**	CD62P	REA	**30.**	MCSP	mIgG1
**12.**	CD146	mIgG1	**31.**	CD44	mIgG1
**13.**	CD41b	REA	**32.**	CD326	mIgG1
**14.**	CD42a	REA	**33.**	CD133/1	mIgG1κ
**15.**	CD24	mIgG1	**34.**	CD29	mIgG1κ
**16.**	CD86	mIgG1	**35.**	CD69	mIgG1κ
**17.**	CD142	mIgG1κ	**36.**	CD45	mIgG2a
**18.**	CD31	mIgG1	**37.**	CD14	mIgG2a
**19.**	CD20	mIgG1			

Supplementary Table 3: List of 92 proteins included in the Inflammation Panel Assay (Olink Bioscience, Uppsala, Sweden).

**1.** Adenosine Deaminase (ADA)	**24.** Fibroblast growth factor 5 (FGF-5)	**47.** Interleukin-17A (IL-17A)	**70.** Oncostatin-M (OSM)
**2.** Artemin (ARTN)	**25.** Fibroblast growth factor 19 (FGF-19)	**48.** Interleukin-17C (IL-17C)	**71.** Osteoprotegerin (OPG)
**3.** Axin-1 (AXIN1)	**26.** Fibroblast growth factor 21 (FGF-21)	**49.** Interleukin-18 (IL-18)	**72.** Programmed cell death 1 ligand 1 (PD-L1)
**4.** Beta-nerve growth factor (Beta-NGF)	**27.** Fibroblast growth factor 23 (FGF-23)	**50.** Interleukin-18 receptor 1 (IL-18R1)	**73.** Protein S100-A12 (EN-RAGE)
**5.** Caspase 8 (CASP-8)	**28.** Fms-related tyrosine kinase 3 ligand (Flt3L)	**51.** Interleukin-20 (IL-20)	**74.** Signaling lymphocytic activation molecule (SLAMF1)
**6.** C-C motif chemokine 4 (CCL4)	**29.** Fractalkine (CX3CL1)	**52.** Interleukin-20 receptor subunit alpha (IL-20RA)	**75.** SIR2-like protein 2 (SIRT2)
**7.** C-C motif chemokine 19 (CCL19)	**30.** Glial cell line-derived neurotrophic factor (GDNF)	**53.** Interleukin-22 receptor subunit alpha-1 (IL-22 RA1)	**76.** STAM-binding protein (STAMPB)
**8.** C-C motif chemokine 20 (CCL20)	**31.** Hepatocyte growth factor (HGF)	**54.** Interleukin-24 (IL-24)	**77.** Stem cell factor (SCF)
**9.** C-C motif chemokine 23 (CCL23)	**32.** Interferon gamma (IFN-gamma)	**55.** Interleukin-33 (IL-33)	**78.** Sulfotransferase 1A1 (ST1A1)
**10.** C-C motif chemokine 25 (CCL25)	**33.** Interleukin-1 alpha (IL-1 alpha)	**56.** Latency-associated peptide transforming growth factor beta 1 (LAP TGF-beta-1)	**79.** T-cell surface glycoprotein CD5 (CD5)
**11.** C-C motif chemokine 28 (CCL28)	**34.** Interleukin-2 (IL-2)	**57.** Leukemia inhibitory factor (LIF)	**80.** T-cell surface glycoprotein CD6 isoform (CD6)
**12.** CD40L receptor (CD40)	**35.** Interleukin-2 receptor subunit beta (IL-2RB)	**58.** Leukemia inhibitory factor receptor (LIF-R)	**81.** T-cell surface glycoprotein CD8 alpha chain (CD8A)
**13.** CUB domain-containing protein 1 (CDCP1)	**36.** Interleukin-4 (IL-4)	**59.** Macrophage colony-stimulating factor 1 (CSF-1)	**82.** Thymic stromal lymphopoietin (TSLP)
**14.** C-X-C motif chemokine 1 (CXCL1)	**37.** Interleukin-5 (IL-5)	**60.** Macrophage inflammatory protein 1-alpha (CCL3)	**83.** TNF-beta (TNFB)
**15.** C-X-C motif chemokine 5 (CXCL5)	**38.** Interleukin-6 (IL-6)	**61.** Matrix metalloproteinase-1 (MMP-1)	**84.** TNF-related activation-induced cytokine (TRANCE)
**16.** C-X-C motif chemokine 6 (CXCL6)	**39.** Interleukin-7 (IL-7)	**62.** Matrix metalloproteinase-10 (MMP-10)	**85.** TNF-related apoptosis-inducing ligand (TRAIL)

**17.**	C-X-C motif chemokine 9 (CXCL9)	**40.**	Interleukin-8 (IL-8)	**63.**	Monocyte chemotactic protein 1 (MCP-1)	**86.**	Transforming growth factor alpha (TGF-alpha)
**18.**	C-X-C motif chemokine 10 (CXCL10)	**41.**	Interleukin-10 (IL-10)	**64.**	Monocyte chemotactic protein 2 (MCP-2)	**87.**	Tumor necrosis factor (Ligand) superfamily, member 12 (TWEAK)
**19.**	C-X-C motif chemokine 11 (CXCL11)	**42.**	Interleukin-10 receptor subunit alpha (IL-10RA)	**65.**	Monocyte chemotactic protein 3 (MCP-3)	**88.**	Tumor necrosis factor (TNF)
**20.**	Cystatin D (CST5)	**43.**	Interleukin-10 receptor subunit beta (IL-10RB)	**66.**	Monocyte chemotactic protein 4 (MCP-4)	**89.**	Tumor necrosis factor ligand superfamily member 14 (TNFSF14)
**21.**	Delta and Notch-like epidermal growth factor related recep (DNER)	**44.**	Interleukin-12 subunit beta (IL-12B)	**67.**	Natural killer cell receptor 2B4 (CD244)	**90.**	Tumor necrosis factor receptor superfamily member 9 (TNFRSF9)
**22.**	Eotaxin-1 (CCL11)	**45.**	Interleukin-13 (IL-13)	**68.**	Neurotrophin-3 (NT-3)	**91.**	Urokinase-type plasminogen activator (uPA)
**23.**	Eukaryotic translation initiation factor 4E-binding protein 1 (4E-BP1)	**46.**	Interleukin-15 receptor subunit alpha (IL-15RA)	**69.**	Neurturin (NRTN)	**92.**	Vascular endothelial growth factor A (VEGF-A)

Supplementary Table 4: Correlations between protein expression levels from primed and non-primed conditions from cells grown in normoxia (left) and hypoxia (right). Proteins above the line are statistically significant with a q-value <0.05. Data is presented with NPX difference, p-values and adjusted p values (q-vales) using a false discovery rate method of 5%.

Difference in Normoxic/Primed vs Normoxic

Protein	Difference	P-value	Q-value
CSF1	3.724	0.000107	0.004274
TGFalpha	2.606	0.000652	0.006865
CCL11	2.667	0.000493	0.006865
CCL3	6.252	0.000688	0.006865
IL13	2.564	0.00198	0.015802
MCP4	3.429	0.003354	0.017247
CXCL10	-0.9942	0.003387	0.017247
MCP2	3.584	0.003458	0.017247
CXCL5	2.708	0.004359	0.019325
TSLP	3	0.005407	0.021576
CCL20	1.519	0.006182	0.022425
CCL4	1.752	0.009573	0.031829
IFNgamma	6.091	0.011497	0.035286
MCP3	2.286	0.0125	0.035624
MMP10	2.437	0.015947	0.04242
IL6	2.219	0.017416	0.04343
LIF	1.677	0.021895	0.051389
Flt3L	1.438	0.028145	0.062389
CXCL11	-0.8993	0.03173	0.065699
BetaNGF	0.8273	0.032932	0.065699
CXCL9	1.247	0.036151	0.068688
MMP1	3.377	0.044704	0.081077
CXCL6	-0.7683	0.050333	0.084833
TWEAK	-0.7422	0.051027	0.084833
CASP8	0.4793	0.053369	0.085177
TRAIL	1.451	0.058343	0.089534
VEGFA	2.791	0.061716	0.09083
GDNF	0.9092	0.06374	0.09083
TNF	3.588	0.06908	0.095045
IL15RA	0.3619	0.107369	0.142801
IL1alpha	1.695	0.133531	0.171867
LAPTGFbeta1	0.3821	0.163333	0.1862
X.4eBP1	-1.504	0.152411	0.1862
STAMBP	-0.8797	0.160673	0.1862
ADA	-0.8529	0.154359	0.1862
CST5	-0.2322	0.191432	0.21217
IL18R1	1.121	0.251311	0.271008
CDCP1	-1.424	0.324902	0.341147
TNFRSF9	0.9176	0.339618	0.347456
MCP1	0.38	0.377593	0.367463
CXCL1	0.4019	0.37241	0.367463
FGF5	-0.4146	0.396812	0.376971
OPG	0.4844	0.413418	0.383613
uPA	-0.6801	0.489101	0.43367
FGF21	-0.2142	0.487206	0.43367
IL8	-0.1714	0.552321	0.479079
HGF	-0.3511	0.684531	0.572024
IL12B	0.0644	0.688149	0.572024
DNER	0.08519	0.736095	0.599392
CD40	0.3371	0.842773	0.672533
PDL1	-0.1568	0.885785	0.692997
NT3	-0.04924	0.94044	0.721607

Difference in Hypoxic/Primed vs Hypoxic

Protein	Difference	P-value	Q-value
CSF1	3.405	0.000002	0.00005
MCP3	3.204	0.000044	0.00061
MCP2	5.55	0.000055	0.00061
MCP4	4.188	0.000177	0.00148
CXCL5	3.353	0.00027	0.00181
IL13	1.345	0.000533	0.00271
IFNgamma	8.305	0.000565	0.00271
OPG	0.6904	0.001101	0.00462
TNF	5.416	0.002989	0.01031
CCL3	6.477	0.00307	0.01031
TGFalpha	1.886	0.004504	0.01224
BetaNGF	0.7525	0.004892	0.01224
CXCL6	-1.14	0.005103	0.01224
LIF	1.777	0.004892	0.01224
CCL11	3.417	0.005601	0.01254
TSLP	1.681	0.006741	0.01332
Flt3L	1.537	0.0065	0.01332
CXCL10	-1.131	0.007153	0.01335
TRAIL	2.136	0.007908	0.01398
IL18R1	0.976	0.01305	0.02192
VEGFA	2.809	0.029564	0.04730
IL1alpha	2.628	0.034249	0.05003
CCL4	1.336	0.033623	0.05003
GDNF	1.304	0.037223	0.05051
TWEAK	-1.316	0.037584	0.05051
IL6	1.581	0.058581	0.07303
CXCL11	-1.055	0.05869	0.07303
CCL20	1.526	0.069599	0.08351
MMP10	1.78	0.072603	0.08411
STAMBP	-0.7282	0.097776	0.1095
CASP8	0.5535	0.104303	0.11305
PDL1	0.8359	0.110889	0.11643
ADA	-0.6468	0.136724	0.1392
HGF	-0.8479	0.193725	0.18880
CD40	0.8398	0.19667	0.18880
IL12B	-0.1342	0.248483	0.23191
MCP1	0.2859	0.447556	0.40642
NT3	0.3725	0.47904	0.42357
CST5	-0.1125	0.542072	0.46701
LAPTGFbeta1	0.2458	0.598704	0.46782
uPA	-0.7977	0.583845	0.46782
CXCL1	0.2282	0.59355	0.46782
DNER	-0.1554	0.598536	0.46782
CDCP1	-0.2375	0.615583	0.47008
CXCL9	0.2274	0.649328	0.48483
TNFRSF9	0.1689	0.670158	0.48950
IL15RA	0.1402	0.753955	0.53899
FGF21	-0.09915	0.794852	0.55639
IL8	-0.055	0.871955	0.59687
4eBP1	-0.2175	0.888211	0.59687
FGF5	-0.00048	0.999141	0.64559
MMP1	-0.01121	0.996219	0.64559

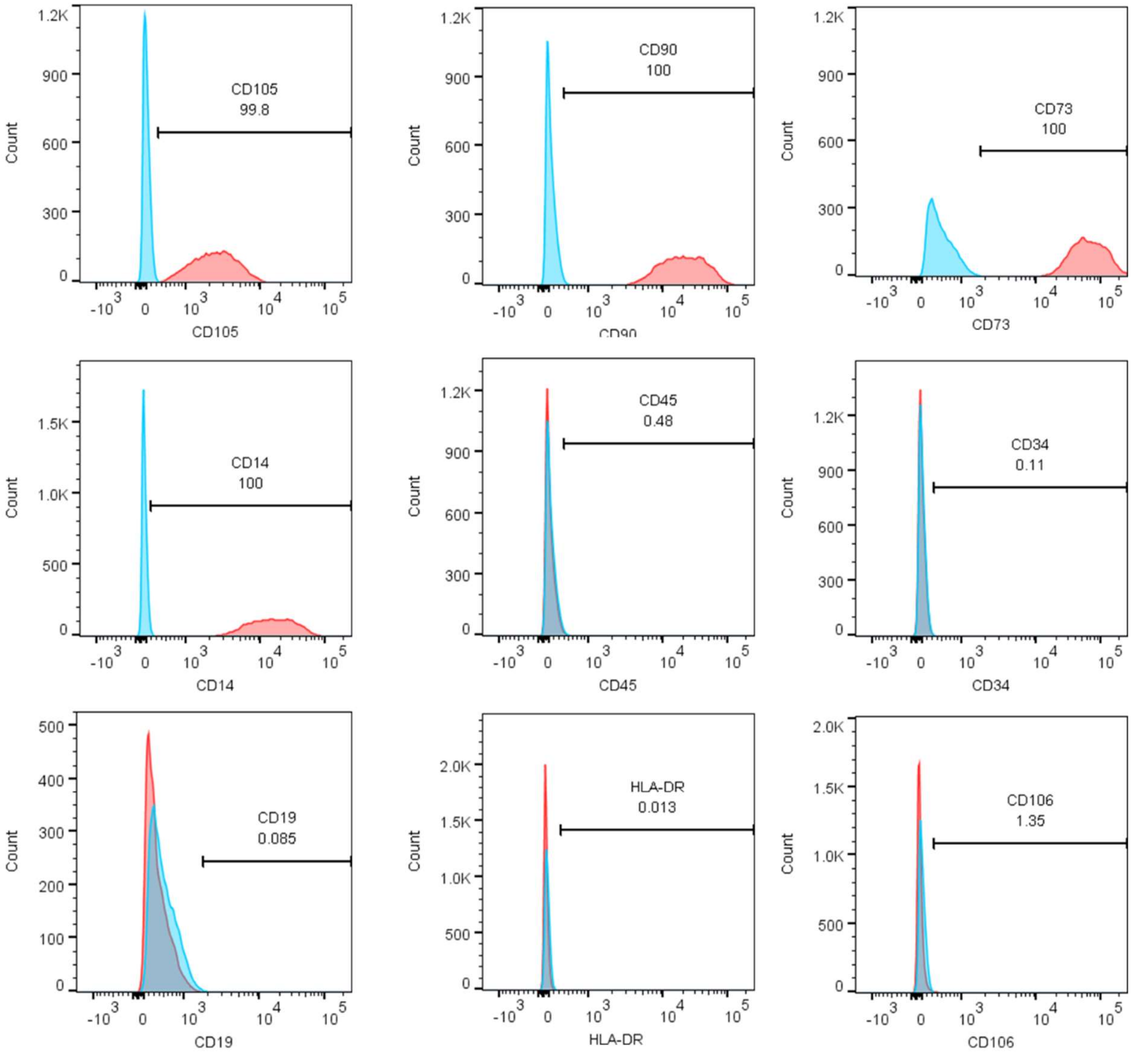

Supplementary Figure 1: Flow charts representing the surface antigen expression on UCMSCs. UCMSCs were positive for CD105, CD90, CD73, CD14 and negative for CD45, CD34, CD19 and HLA-DR.

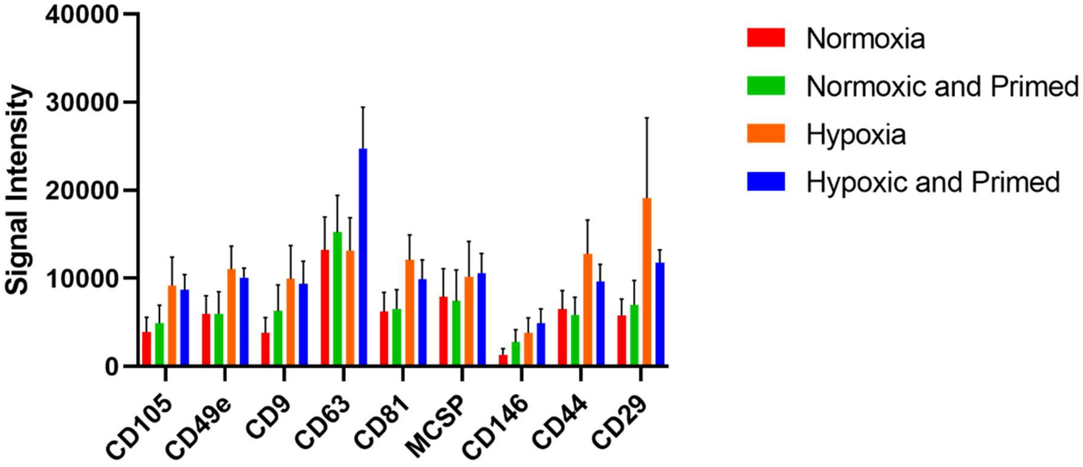

Supplementary Figure 2: Range of surface markers expressed on EVs (n=4) grown in four conditions. There were no statistical differences between conditions. Errors bars ± SEM.

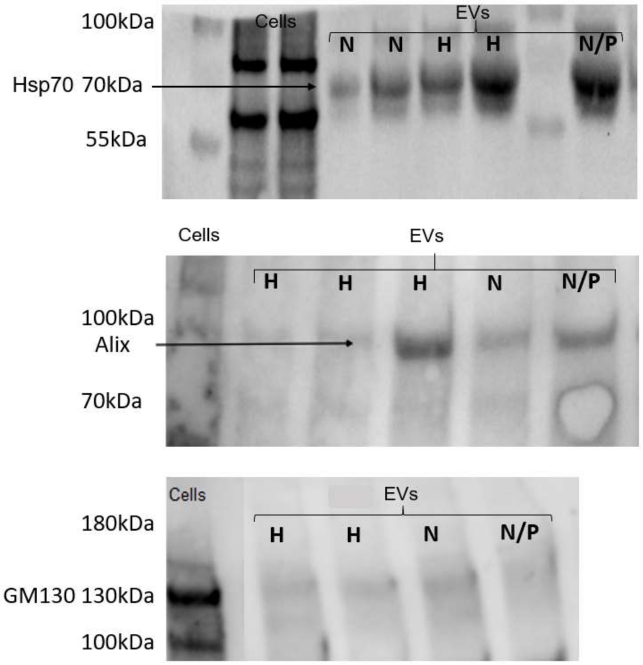

Supplementary Figure 3: Immunoblotting identification of Hsp70 and Alix in cells and EVs from normoxic (N), hypoxic (H) and normoxic/primed (N/P) conditions. Positive expression of GM130 in cells.
